# Adherence to dietary recommendations mediates the effect of affective temperaments on infertility treatment outcomes

**DOI:** 10.1038/s41598-024-63343-x

**Published:** 2024-05-31

**Authors:** Georgina Szabo, Judit Szigeti F., Miklos Sipos, Szabolcs Varbiro, Xenia Gonda

**Affiliations:** 1 Doctoral School of Mental Health Sciences, Semmelweis University, Budapest, Hungary; 2 Institute of Behavioral Sciences, Semmelweis University, Budapest, Hungary; 3 Department of Obstetrics and Gynecology, Semmelweis University, Budapest, Hungary; 4 Department of Psychiatry and Psychotherapy, Semmelweis University, Gyulai Pal utca 2, Budapest, 1085 Hungary; 5 NAP3.0-SE Neuropsychopharmacology Research Group, Hungarian Brain Research Program, Semmelweis University, Nagyvárad tér 4., Budapest, 1085 Hungary

**Keywords:** Psychiatric disorders, Psychology

## Abstract

Affective temperaments have been shown to robustly affect infertility treatment success. However, identification of possible mediating factors through which they exert their influence is still lacking. A growing number of results suggest that adherence to recommended treatments may be such a mediator, on the one hand, because affective temperaments are known to influence adherence and, on the other hand, because non-adherence negatively influences the treatment outcome. Recommended treatment of infertility involves, beyond medications, dietary and lifestyle changes. The aim of this retrospective cohort study was to evaluate whether adherence to physician-prescribed diet and physical activity recommendations mediates the effect of affective temperaments on infertility treatment outcomes. Among 308 women who underwent infertility treatment in an Assisted Reproduction Center, affective temperaments, adherence to diet, adherence to physical exercise, and infertility treatment success (clinical pregnancy) were assessed besides detailed medical history and demographic parameters. Associations between affective temperaments, adherence to diet and recommended physical activity, and assisted reproduction outcomes were analyzed using generalized linear models and causal mediation analysis. Adherence to physical activity didn’t have an effect, but diet adherence increased the odds of infertility treatment success by 130% suggesting its role as a potential mediator. Based on causal mediation analysis, higher depressive and anxious temperament scores were directly associated with 63% and 45% lower odds of achieving clinical pregnancy, respectively, with effects not mediated by diet adherence. Higher irritable temperament scores indirectly decreased the odds of achieving clinical pregnancy by 14%, mediated by diet adherence; while higher cyclothymic temperament scores decreased the odds of achieving clinical pregnancy both directly by 51% and indirectly, mediated by diet adherence by 11%. Our results suggest that diet adherence mediates the mechanism by which irritable and cyclothymic affective temperaments influence IVF treatment success. Since adherence is a modifiable risk factor of infertility treatment success, screening for affective temperaments may help to identify potentially high-risk non-adherent patient groups and offer patient-tailored treatment, which may help increase the chances of a successful pregnancy and live birth in women undergoing IVF treatment.

## Introduction

Infertility directly affects one in six people of reproductive age in their lifetime^1^, and its prevalence is steadily increasing^2^, with a parallel rise in the number of assisted reproductive procedures^3^. Infertility may be caused by a number of factors, from somatic^1^, and lifestyle^4^ to psychological^5^, and therefore, effective treatment of infertility is a complex and often expensive, lengthy, and exhaustive process^6^, that requires extreme adherence, not only in terms of medication or compliance with treatment protocols, but also in terms of following the diet or physical exercise plan recommended by the doctor as part of the treatment plan^7–12^. Infertility treatments have a cumulative success rate of less than 50%^13^. In addition, infertility treatment can often be associated with depression and anxiety caused by chronic stress and the constant alternation of hope and disappointment^14^, making the importance of psychological screening prior to infertility treatment essential to identify patients at risk of emotional problems during subsequent treatment^15^. However, from a screening perspective, it would be at least as important to investigate the psychological determinants of patient non-adherence to treatment protocols, as the average rate of medication adherence in infertility treatment is only around 50%^8^, and patients do not seem to follow recommendations for lifestyle behavior modification during infertility treatment either^7–12^, which seriously compromises the effectiveness of treatment and is, therefore, a critical issue for population health, both in terms of quality of life and health economics^16^.

Therapy adherence is a highly complex process that involves a number of factors of both a dynamic and static nature, including a complex pattern of factors related to the patient, the treatment, the doctor-patient relationship and the environment, many of which are underpinned by the psychological characteristics of the patient^17^. Moreover, in contrast to the more tangible barriers to treatment adherence, such as lack of psychoeducation, polypharmacotherapy, low frequency of follow-up, to name but a few, psychological barriers are more challenging and complex, and thus more difficult to identify and address^18^. Important components -or even determinants- of these psychological barriers include patients' personality and temperament factors, which have been less studied in this context in the past, although they are strongly linked to adherence-related behaviours by identifying emotions, cognitions, attitudes and reactions^19^.

Affective temperaments (depressive, cyclothymic, hyperthymic, irritable, and anxious) represent vulnerability factors towards the development of various mental disorders^20,21^ and somatic diseases^22–33^ and affect the long-term course and treatment outcome of several of these conditions^20,29,31,34^. Unlike other models which universally describe basic personality traits, the model of affective temperaments has been developed based on observation of affective disorder patients and their first-degree relatives and focuses on emotional reactivity and related cognitions and behaviors. We chose to investigate this model in part due to its focus on sub- or maladaptive emotional reactions to everyday situations and events which may yield enhanced usefulness when trying to understand barriers of profiting from medical treatment, and also because previously this model has not been investigated in this context. In a previous study, we reported that affective temperaments also affect infertility treatment success^35^, and we discussed that the necessary next step is to identify the processes and factors through which they exert their influence, and which may be modifiable with psychological interventions and patient-centered, targeted therapy. A growing number of studies suggest that the role of affective temperaments in prognosis and treatment success in somatic illnesses in part may be related to their impact on complying with treatment recommendations, on the one hand, because affective temperaments influence the degree of adherence^36^, and on the other hand, because non-adherence negatively influences treatment outcome^16^. Since non-adherence to recommendations about following a specific diet or exercise plan may be predictable by affective temperaments, and also they present modifiable risk factors compromising infertility treatment success, diet- and sport-adherence might be potential mediators of the already proven affective temperament – infertility treatment success relationship.

The aim of the present study was to evaluate whether adherence to diet and physical activity recommendations mediates the effect of affective temperaments on infertility treatment outcomes.

## Methods

### Study population

Women presenting for first appointment at the Assisted Reproduction Center of the Department of Obstetrics and Gynecology of Semmelweis University between November 2019 and November 2021 were contacted, and those who agreed to participate and met inclusion criteria were involved in the study. Inclusion criteria were as follows: women between 18 and 45 years by the time of first appointment (considering that assisted reproduction is supported in Hungary between the ages of 18 and 45), who have attempted fertility within the past three years for more than a year, and doctor’s recommendation of having diet and regular physical activity as part of their treatment so that adherence can be interpreted and evaluated. Also, it was necessary to have a good command of the Hungarian language, willingness to participate in the study, provide informed consent, and complete the questionnaire. Patients having fertility preservation due to cancer treatment or patients requiring assisted reproductive procedure solely due to known male but not female infertility, or patients visiting assisted reproduction centre with no intention of getting pregnant were excluded. Number of previous spontaneous or assisted pregnancies and number of children did not influence inclusion/exclusion in the study.

Of the 578 patients presented in our previous study^35^ , 308 of them had both diet and sports recommendations in their treatment plan, so they were included in this study. Demographic, anthropometric, psychometric, and medical data of all patients were recorded by self-assessment questionnaires or were abstracted from the medical records. All patients agreed to data retrieval and analysis and provided written informed consent prior to filling out the questionnaires. The study was approved by the Scientific and Research Ethics Committee of the Medical Research Council, the Hungarian Ministry of Health (IV/1568- 1 /2022/EKU), and was carried out in accordance with the tenets of the Declaration of Helsinki.

### Evaluation of affective temperaments

Affective temperaments were measured by the Temperament Evaluation of Memphis, Pisa, Paris and San Diego (TEMPS-A) auto-questionnaire. TEMPS-A is a 110-item self-report instrument developed to assess affective temperaments in cyclothymic (a biphasic temperament associated with rapid mood fluctuation and swings, emotional instability, and also rapid changes in self-esteem and confidence), depressive (proneness to possess a generally lower mood in addition to adhering more to rules and daily routines, being sensitive to criticism and guilt), anxious (characterised by out-of-control worrying and fearfulness and a general apprehension of danger, worrying about potential unluck, and consequential hypervigilance), irritable (related to the parallel presence of depressive and hyperthymic characteristics contributing to being sceptic and critical, prone to anger and complaining), and hyperthymic (being optimistic and outgoing, self-confident, with less need for sleep and less prone to fatigue) subscales, requiring “yes” (score 1) or “no” (score 0) answers^37–39^.

The questionnaire showed good to excellent internal reliability of the scales in the Hungarian normative population in the validation study^38^: depressive (21 items, Cronbach’s alpha = 0.63); cyclothymic (21 items; Cronbach’s alpha = 0.81); irritable (21 items; Cronbach’s alpha = 0.79); anxious (26 items; Cronbach’s alpha = 0.84); and hyperthymic (21 items; Cronbach’s alpha = 0.78). In our current sample, very similar or better internal reliability of the scales was confirmed: depressive (21 items, Cronbach’s alpha = 0.64); cyclothymic (21 items; Cronbach’s alpha = 0.84); irritable (21 items; Cronbach’s alpha = 0.79); anxious (26 items; Cronbach’s alpha = 0.87); and hyperthymic (21 items; Cronbach’s alpha = 0.78).

### Assisted reproduction techniques

The techniques used were heterogeneous based on the patients' needs and associated medical conditions. In most cases, ovarian stimulation was applied according to one of the standard protocols^40^ followed by either traditional in vitro fertilization (IVF) or intracytoplasmic sperm injection (ICSI), supplemented as necessary with oral medication or other kind of treatment of the presumed problems behind the infertility. Due to the heterogeneity of the protocols and the fact that, according to a comprehensive meta-analysis, particular techniques have not been found to be predictors of treatment outcome^41^, in this study, infertility treatment was considered a constant.

### Evaluation of treatment success

Infertility treatment success was defined as clinical pregnancy (confirmed by ultrasound with the presence of amniotic sac, embryo, and fetal heartbeat) following infertility treatment, as reported by the patients.

### Evaluation of adherence

Although the patients to whom the doctor recommended both diet and exercise were all affected by some kind of metabolic problem on a clinical or subclinical level, their diagnosis was heterogeneous. It also implies that the patients did not receive uniform but individualized recommendations in terms of both diet and exercise; therefore, their adherence had to be assessed accordingly. In most cases, the diet consisted of a personalized daily or per-meal carbohydrate intake plan recommended by their dietician, while the recommended physical activity usually was some kind of dynamic exercise 3–5 times a week for at least 40 min. In order to be able to establish adherence to these personalized recommendations, we examined the difference between the proposed and the current diet and exercise as follows:

Whether patients have received recommendations to adhere to a diet or physical activity as part of their treatment plan was assessed both as self-reported and as retrieved from the medical records. Adherence to diet and physical activity was assessed by 7-item Likert scales derived from the Summary of Diabetes Self-Care Activities (SDSCA) questionnaire^42^ and adapted for a retrospective setting. Regarding diet adherence, patients were asked whether their treating physician recommended adopting a special diet/meal plan during their assisted reproduction treatment, and in case of a positive answer, they were asked how many days out of seven days in an average week they were able to follow the meal plan recommended by their doctor during their assisted reproduction treatment. Those patients were considered adherent to dietary recommendations, who recalled and reported following their physician’s diet recommendations every day. Regarding sports adherence, participants were asked if their treating physician recommended regular exercise during their assisted reproduction treatment, and if yes, how many days a week did their doctor recommend exercise, and on how many days in an average week were they able to do the recommended exercise. Those patients were considered adherent to physical activity recommendations, who recalled and reported at least the recommended number of weekly workouts. Those who self-reported that they had not received medical recommendations to follow a diet or to have regular physical activity, but their clinical record indicated otherwise, were treated as non-adherent.

### Evaluation of covariates

Age at the time of the first appointment was calculated from the difference between the date of the first appointment and the date of birth. The baseline BMI at the first appointment was retrieved from clinical records. The fact of previous miscarriages as well as patients’ diagnosed somatic diseases and mental disorders were self-reported by the patients.

### Statistical analysis

Of the 578 patients presented in our previous study^35^, 308 had dietary and sports recommendations in their treatment plan, so they were included in this current study. The main characteristics of this subgroup of 308 patients were compared with the entire population of 578 patients using the Wilcoxon rank sum test for continuous variables and Pearson's Chi-squared test for categorical values. Relying on the similarity of the two populations, in this analysis, we work with the previously identified covariates; the other examined variables are not reported here again. Continuous variables are expressed as mean ± standard deviation (SD) and range; categorical variables are expressed as numbers and percentages. Between-group differences in descriptive characteristics were detected using the Wilcoxon rank sum test for continuous variables and Pearson's Chi-squared test for categorical values. Univariate linear regression analyses were performed to examine the relationship between infertility treatment success as the response variable and diet or sport adherence as possible predictor variables. Both models were adjusted for age, BMI, and previous miscarriage as already known confounders. After that, causal mediation analysis was applied in order to examine whether any of the adherence variables that significantly predicted infertility treatment success based on the univariate analysis mediates the effect of affective temperaments on infertility treatment success. Causal mediation analysis was run with affective temperaments as exposure, infertility treatment success (clinical pregnancy) as response, and adherence as mediator variables, also adjusted for age, BMI, and previous miscarriage. The results are presented as odds ratios (OR) and 95% confidence intervals (CI). The significance threshold was set to p < 0.05 in all analyses. All calculations were performed using R Statistical Software (Vienna, Austria version 4.2.2).

### Sample size estimation

Since power estimation tools are lacking for mediation analysis, we used published empirical guidelines in determining the sample size necessary to conduct mediational studies with 80% statistical power^43^. Calculating with medium effect sizes for exposure on mediator and mediator on outcome, with an alpha of 0.05 and power of 80%, the suggested minimum sample size range needed was between 116 and 178.

## Results

### Descriptive statistics

The analysis included 308 women (22–46 years of age) who underwent infertility treatment within the last three years and whose treatment plan included both diet and regular physical activity. Since this study is a continuation of our previous study^35^, as a first step, the main characteristics of this subgroup of 308 patients were compared with the entire population of 578 patients, and no statistically significant difference was found between the demographic, anthropometric, psychometric, and medical characteristics of the two groups except for BMI (25.83 ± 5.87 vs. 24.24 ± 4.90 kg/m^2^ (W = 103,380, *p* < 0.001)) and diagnosed metabolic disorder (79% vs. 42% (χ^2^ = 108, p < 0.001)) which is a natural consequence of the fact that this group includes the patients who needed to diet and exercise.

In this current subgroup, 201 (65%) of the participants were primarily infertile, 45 (15%) already had at least one child from a previous pregnancy, while 62 (20%) did not have children yet but already achieved pregnancy which ended in miscarriage(s). The mean age of our cohort was 35.51 ± 4.78 years, and the mean BMI was 25.83 ± 5.87 kg/m^2^. The probable co-factors associated with infertility were highly variable among the patients, the most typical of which included various problems of metabolic disorders (79%), thyroid disorders (44%), endometriosis (9.1%), or the combination of these. The applied assisted reproduction treatments were diverse. In most cases, ovarian stimulation was applied according to one of the standard protocols^40^ followed by either traditional in vitro fertilization (IVF) or intracytoplasmic sperm injection (ICSI), supplemented as necessary with medications or other kinds of treatment for the presumed problems behind the infertility. All of the patients had to follow a specific diet and physical activity schedule as part of their treatment, but only 43% of the patients adhered to the diet, and 38% of the patients adhered to the physical activity recommended by their doctor. The applied infertility treatment resulted in clinical pregnancy in 116 out of 308 cases (38%).

The mean age of patients who successfully became pregnant after the treatment was approximately two years less, as compared to those who failed to conceive after treatment (34.36 ± 4.61 vs 36.20 ± 4.76 years (W = 13,592, *p* = 0.001)). No statistically significant difference could be observed regarding BMI and previous miscarriage. Regarding affective temperaments, the mean score of cyclothymic and anxious temperaments proved to be significantly lower in the pregnant cohort compared to the non-pregnant one (4.22 ± 4.15 vs 5.21 ± 4.16 points (W = 12,985, *p* = 0.014) and 6.80 ± 5.20 vs 8.20 ± 5.54 points (W = 12,782, *p* = 0.03), respectively). The proportion of those patients who adhered to the diet plan was significantly higher in the group that successfully became pregnant (55% vs 35% (χ^2^ = 12, p < 0.001)). The rate of adherence to physical activity did not differ significantly in the two groups. Demographic parameters, adherence, and scores in the different scales of affective temperaments are summarized in Table 1.

**Table 1:** TEMPS-A scores, adherence, and previously identified infertility risk factors as covariates in patients with successful and unsuccessful infertility treatment.

Characteristics		Treatment success (clinical pregnancy)		Test statistic	p-value
	Total	(−)*	( +)*		
Number	308	192 (62%)	116 (38%)	*–*	*–*
Covariates					
Age (years)	35.51 (4.78) [22.00, 46.00]	**36.20 (4.76) [22.00, 45.00]**	**34.36 (4.61) [24.00, 46.00]**	W = 13,592	**0.001**
BMI (kg/m2)	25.83 (5.87) [16.50, 55.60]	26.36 (6.26) [16.50, 55.60]	24.95 (5.07) [17.40, 42.40]	W = 12,430	0.088
Previous miscarriage	102 (33%)	56 (29%)	46 (40%)	χ^2^ = 3.6	0.058
Affective temperaments					
Cyclothymic	4.84 (4.18) [0.00, 19.00]	**5.21 (4.16) [0.00, 18.00]**	**4.22 (4.15) [0.00, 19.00]**	W = 12,985	**0.014**
Depressive	7.43 (2.95) [0.00, 17.00]	7.69 (2.95) [2.00, 17.00]	7.00 (2.93) [0.00, 15.00]	W = 12,523	0.066
Anxious	7.68 (5.45) [0.00, 22.00]	**8.20 (5.54) [0.00, 22.00]**	**6.80 (5.20) [0.00, 22.00]**	W = 12,782	**0.03**
Irritable	4.12 (3.54) [0.00, 16.00]	4.27 (3.66) [0.00, 16.00]	3.87 (3.34) [0.00, 15.00]	W = 11,767	0.4
Hyperthymic	10.06 (4.09) [0.00, 20.00]	9.76 (4.06) [1.00, 20.00]	10.57 (4.12) [0.00, 20.00]	W = 9,699	0.057
Adherence					
Adhered to diet	131 (43%)	**67 (35%)**	**64 (55%)**	χ^2^ = 12	** < 0.001**
Adhered to physical activity	117 (38%)	70 (36%)	47 (41%)	χ^2^ = 0.51	0.5

Continuous variables are expressed as mean ± standard deviation (SD) and range, categorical variables are expressed as numbers and percentages. The p-values are calculated by Wilcoxon rank sum test and Pearson’s Chi-squared test. The bold values in the table represent significant findings.

*BMI* body mass index.

### Regression analyses

Based on univariate regression analysis, adherence to physical activity didn’t (OR = 1.229 (p = 0.413)), but diet adherence increased the odds of infertility treatment success by 130% (OR = 2.299 (p = 0.001)), and thereby, possibly could be a potential mediator between affective temperaments and infertility treatment success. Table 2 presents the results of the univariate logistic regression analyses of diet and sport adherence on assisted reproduction treatment success (clinical pregnancy), adjusted for age, BMI, and previous miscarriage as covariates.

**Table 2:** Results of univariate logistic regression analyses of diet and sport adherence on assisted reproduction treatment success (clinical pregnancy).

Variable	OR	95% CI		p
Diet adherence	**2.299**	**1.408**	**3.755**	**0.001**
Sport adherence	1.229	0.750	2.012	0.413

The table shows the results of univariate logistic regression analyses using diet and sport adherence as exposure variables, and infertility treatment success (clinical pregnancy) as outcome variable. Models are adjusted for age, BMI, and previous miscarriage. The bold values in the table represent significant findings.

*OR* odds ratio, *CI* confidence interval.

### Causal mediation analyses

**Figure 1:**
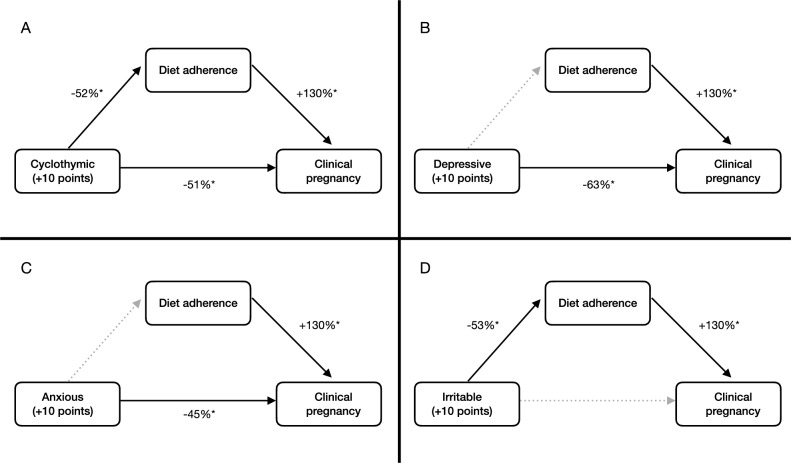
Mediation analyses results. (**A**): Direct and indirect effects of cyclothymic affective temperament on infertility treatment success, the latter mediated by diet adherence. (**B**) and (**C**): Direct effect of depressive and anxious affective temperaments on infertility treatment success, not mediated by diet adherence. (**D**): Indirect effect of irritable affective temperament on infertility treatment success, mediated by diet adherence. Solid arrows and starred effect sizes indicate significant effects, while dotted arrows indicate nonsignificant pathways.

Based on causal mediation analysis, higher depressive and anxious temperament scores were directly associated with a lower likelihood of achieving clinical pregnancy (direct effect: OR = 0.369 (p = 0.023), and OR = 0.554 (p = 0.013), respectively), with effects not mediated by diet adherence (mediation effect: OR = 0.917 (p = 0.259), and OR = 0.954 (p = 0.284), respectively) (Fig. 1B,C); higher irritable affective temperament scores indirectly decreased the likelihood of achieving clinical pregnancy, mediated by diet adherence (mediation effect: OR = 0.885 (p = 0.027), direct effect: OR = 0.692 (p = 0.307)) (Fig. 1D); while higher cyclothymic affective temperament scores decreased the likelihood of achieving clinical pregnancy both directly (OR = 0.492 (p = 0.027)) and indirectly, mediated by diet adherence (OR = 0.891 (p = 0.034)) (Fig. 1A). Hyperthymic affective temperament had no statistically significant effect on infertility treatment success. Table 3 presents the results of the causal mediation analyses.

**Table 3:** Results of causal mediation analysis of affective temperaments on clinical pregnancy outcome with diet adherence as mediator (n = 308).

	Mediation effect				Direct effect				Total effect			
Exposure	OR	95% CI		p	OR	95% CI		p	OR	95% CI		p
Cyclothymic	**0.891**	**0.801**	**0.991**	**0.034**	**0.492**	**0.262**	**0.921**	**0.027**	**0.438**	**0.234**	**0.821**	**0.010**
Depressive	0.917	0.788	1.066	0.259	**0.369**	**0.157**	**0.870**	**0.023**	**0.338**	**0.142**	**0.807**	**0.015**
Anxious	0.954	0.876	1.040	0.284	**0.554**	**0.348**	**0.884**	**0.013**	**0.529**	**0.330**	**0.849**	**0.008**
Irritable	**0.885**	**0.795**	**0.986**	**0.027**	0.692	0.341	1.404	0.307	0.612	0.300	1.251	0.178
Hyperthymic	0.992	0.886	1.111	0.893	1.764	0.980	3.173	0.058	1.750	0.962	3.184	0.067

The table shows the results of mediation analysis using 10 points of change in TEMPS-A scores, diet adherence and clinical pregnancy as exposure, mediator and outcome variables, respectively. Population: patients whose treatment plan includes both diet and sport recommendation (n=308). The models are adjusted for age, BMI, and previous miscarriage. The bold values in the table represent significant findings.

*CI* confidence interval, *OR* odds ratio.

## Discussion

In our study, we demonstrated that affective temperaments robustly influence the chance of achieving a pregnancy via fertility treatments through their association with adherence to dietary but not physical activity recommendations as part of the treatment plan. We found that while hyperthymic temperament had no effect, the other four temperaments lowered the chance of success, and irritable and cyclothymic temperaments exerted their effect completely or in part via the mediation of diet adherence.

Although it is increasingly well-known that affective temperaments influence the success of somatic treatments, little is known about what mediates this relationship, which significantly limits their application in clinical practice. As affective temperaments are not modifiable themselves, identifying the specific and potentially modifiable factors mediating their effects would help exploit their potential for psychological screening to personalize treatment components and provide psychological support to ensure their successful execution. This is important because affective temperaments are easy to screen with the validated TEMPS-A self-report instrument, and thus, they could be used to identify patients for whom personalized psychological, educational interventions or other means to promote dietary adherence could improve the success rate of assisted reproduction while their lack of association with adhering to physical activity recommendations also shows that other psychological factors helping to tackle of this particular problem should be identified. Our findings concerning the fact that the effect of affective temperaments on the success of fertilization treatments is particularly important because diet adherence had a very marked effect on success, whereas BMI alone did not, and neither did exercise.

### Unmet needs in increasing the success rate of fertility treatments

It is estimated that one in six people of reproductive age will be directly affected by infertility in their lifetime^1^. Between 1990 and 2017, the prevalence of infertility increased by 0.3–0.4% per year based on the statistics of 195 countries^2^. In parallel, the number of assisted reproductive procedures is also steadily increasing^3^. The treatment of infertility is complex and multifaceted, and the term medically assisted reproduction includes not only in vitro treatments but also in vivo methods, ranging from lifestyle changes that do not require major medical intervention, to drug or surgical treatment of various underlying somatic diseases, to drug stimulation of follicular maturation, to insemination^6^. In addition to the time and effort required for screening and treatment, there are several potential stumbling blocks at any point in the process. To mention just the most common ones: ovaries may become under- or over-stimulated, retrieved eggs may be immature, sperm collection may be problematic, fertilization may fail, division may stop, embryos may not survive freezing, and implantation may fail. Finally, one-fifth of induced pregnancies end in miscarriage^13^. The average success rate of assisted reproduction per cycle of intervention in clinical pregnancy is 26%, and the rate per embryo transfer is around 34%^44^. In other words, the chances of a treatment failing are higher than those of succeeding. According to German data, 10 out of 12 couples who undergo in vitro fertilization will go on to implantation, three will become pregnant, and two will take home a baby from hospital^45^. The cumulative pregnancy rate, i.e., the rate of multiple pregnancies achieved with multiple interventions, is naturally higher, and the cumulative survival rate for patients under 40 years of age is around 50% for six implantations^13^. Among the factors influencing success, previous literature suggests that age (mainly female), duration of subfertility, basal FSH level, and number of eggs retrieved are the most important^46^. Other hormone levels, body mass index, sperm quality, and various genetic and environmental factors are also involved, even in an interrelated way^47^. Unfortunately, most of these factors are not modifiable risk factors for infertility.

### The potential role of psychological factors and interventions in increasing the success rate of infertility treatments

The influence of psychological characteristics arises after or in complex relation to the above biological and social factors. Infertility itself and the often very lengthy process of infertility treatment are highly stressful psychologically, and therefore, psychological support should be an essential part of the treatment. However, it is also becoming increasingly apparent that the role of psychological factors is much broader than simply providing mental support as we become more aware that personality and psychological factors have a significant impact not only on the development of various somatic conditions and illnesses but also on their long-term course and the success of their treatment^29,31,34,48–50^. Given the potential of these factors as modifiable risk factors for the success of somatic interventions, it is important to explore the possible mechanisms through which these psychological factors contribute, directly or indirectly, to the success of infertility interventions and to understand in more detail which of these factors are indeed easily modifiable and screenable.

### The role of affective temperaments in the success of fertility treatment and potential clinical implications

As described earlier, affective temperaments, for example, are such that they are likely to affect the outcome of medical interventions and are easily screened, and while they themselves cannot be modified, they do affect adherence, at least in terms of medication for sure, which can be modified. The effect of affective temperaments on treatment outcomes has also been shown in relation to infertility treatments, but what mediates this effect, which could be the aforementioned adherence or various other psychological or behavioral factors, has not been investigated yet.

In the present study, we investigated adherence, whether exercise and dietary adherence mediate the above-mentioned effects, both because they are necessary and very common in infertility treatment and because, although they require considerable effort, they can be helped in appropriate ways, such as education, support, coaching, etc., thus contributing to the increased success rate of the treatment. Our results showed that, while adherence to physical activity does not, diet adherence mediates the effect of certain affective temperaments. It is also remarkable that the effect of depressive and anxious temperaments on clinical pregnancy are not mediated by dietary adherence, cyclothymic is partly mediated, while in case of irritable temperament only this mediated effect is present, and the temperament itself does not have a direct influence on treatment success. Also, hyperthymic temperament showed no effect on treatment outcomes. We can only hypothesize about the exact temperament-adherence-outcome mediating mechanism, but overall, it is likely that depressed and anxious temperaments may result in lower pregnancy rates for physiological reasons, whereas such patients are very careful to comply with all such instructions. Cyclothymic and irritable temperaments are the ones who are more unstable, and that can make it difficult to sustain commitment and persistence, especially when facing difficulties, so in their case, the adherence problem is understandable. In the case of the hyperthymic temperament, it is also understandable that there are basically no psychological processes that can vegetatively harm the ability to conceive, but at the same time, their emotional balance makes these patients very likely to be able to adhere to medical prescriptions. Further studies are needed to elucidate exactly how cyclothymic and irritable temperament affect diet adherence, but it has already been demonstrated that these temperament types also negatively affect medication adherence^36^ explained by the typical characteristics of these affective temperament types, such as both temperament types reacting less favorably to disturbing events^51^, or cyclothymic temperaments being characterized by a higher degree of intolerance to subjective pain and a tendency to an increased emotional response to intense stressful and painful experiences^39,52^, which is also associated with hopelessness^53^. These temperaments would, therefore, make it difficult to bear the burden of long-term medical intervention, to accept the fact of clinical condition or, in this case, infertility, and to adhere to the necessary treatment for a long period, even years, may it be medication, diet or other lifestyle adjustments.

Therefore, our results offer important implications for clinical practice. Psychological work with patients with somatic conditions can be multifaceted, and meta-analytic data suggest that, in general, psychosocial support accompanying assisted reproduction improves both mental health and pregnancy outcomes for participants^54–60^, Beyond standard psychological monitoring and support of these patients which could be preventive in terms of the development of emotional adjustment disorders as a consequence of stress and psychological burden associated with infertility and infertility treatment, psychological interventions could also significantly support patients’ ability to adhere to dietary recommendations in those patients whose affective temperamental profile suggest that their lower dietary adherence may jeopardize treatment success. While affective temperaments cannot be modified by psychotherapy, they can be used to identify patients at risk of less intervention success, and by understanding the factors that mediate this lower success rate in case of given affective temperaments, extra care, attention, and targeted intervention could focus on these mediatory factors. There are already specific protocols designed to increase dietary adherence, such as the I.M.P.R.O.V.E. strategic, systematic approach, which has been proposed to improve adherence to advised lifestyle for PCOS patients^61^, and applying such protocols to patients who have been screened at the initiation of the treatment to be more likely less adherent to dietary recommendations would likely increase treatment success and thus would certainly be far more cost-effective than the cost of unsuccessful assisted reproduction interventions. Besides identifying the role of dietary adherence specifically mediating the effect of cyclothymic and irritable temperaments on infertility treatment success, our study also sheds light on the need for identifying how psychological factors can be used to screen for behavioral factors mediating their effect in impacting the success of interventions. The aim of such studies is to identify those specific behaviors or psychological processes that could be either modified or alternative interventions can be identified, and prescribed for those who, due to the unchangeable nature of these processes, would be unable to comply with or benefit from the original interventions. In our case, if the temperamental constellation makes it difficult for patients to steadfastly follow through with a diet or exercise plan or adhere in a sustained way to other elements of treatment, either different intervention approaches or specific counseling, education, or support could be prescribed for such patients. This emphasizes the importance of personality screening prior to the intervention.

The strengths of the present study include its fair sample size and its gap-filling nature in analyzing the mediators of the relationship between affective temperaments and the success of assisted reproductive treatment, thus helping to identify modifiable infertility risk factors that can be applied in clinical practice. Our study also has some limitations. First, it is limited by the self-report nature of the questionnaires and the retrospective design, due to which the assessment of adherence is mostly based on the patient's memory. As a consequence, it is liable to recall bias, and it can also be distorted by how much patients dare to admit to the interviewer, to what extent they try to comply with the interviewer’s expectations, or how much they want to attribute the failure of infertility treatment to the investigated factors, which may also be related to personality and temperament. However, we must also add that data retrospectively provided in our study regarding diet and physical activity are relatively reliable (as validated based on medical records). Second, we defined an adherent as someone who fully adhered to the treatment plan, although we are aware that there are studies that are more permissive in their choice of cutoff points. However, there is currently no formally validated questionnaire for either diet or exercise adherence with a commonly used cutoff point. In addition, because of the retrospective setting, we considered it important to have a more rigorous classification to balance possible inaccuracies due to memory. Next, in relation to the retrospective design, it should also be noted that affective temperaments were measured after and not before treatment; however, given the temporal stability of affective temperaments over the life span, this has been unlikely to influence our results. Due to the retrospective nature of the study we could not consider several factors related to infertility treatment as potential confounders, including data on the IVF/ICSI treatments: basal hormonal biomarkers of ovarian reserve (FSH, AMH, AFC), type of protocol, type of gonadotropins, starting dose, total dose, number of dominant follicles, number of oocytes retrieved, number of mature oocytes, fertilization rate, number of embryos transferred, stage of embryos transferred, which may influence pregnancy rates. However, our study is psychological in nature and aimed at identifying those behavioral and emotional/psychological factors which contribute to treatment success in a general infertility treatment population. We also did not include type of infertility treatment in our regression model, in part due to the above reason and also because a recent comprehensive metaanalysis^41^ found that particular IVF techniques did not predict treatment outcome allowing us to consider the type of infertility treatment as constant. Finally, although not specifically a limitation, it is worth mentioning that although BMI is a well-known modifiable risk factor for infertility, BMI can only indirectly and imprecisely predict positive changes. On the one hand, because as body fat increases and muscle mass decreases with age, changes in height, weight, and BMI do not necessarily correspond to proportional changes in body fat or muscle mass, so BMI does not accurately reflect visceral fat accumulation, which is the most common cause leading to metabolic and clinical consequences of obesity^62^. On the other hand, it is increasingly recognized that a state of “metabolically healthy” obesity exists, whereby some individuals are immune to the metabolic complications of obesity, possibly due to visceral adiposity and residual insulin sensitivity, which may also influence infertility treatment success, and BMI does not necessarily predict it well^63^. Despite all this, BMI is the most commonly used, cheapest and easiest to measure predictor of overweight and obesity^64^, which is an important modifiable risk factor for infertility.

## Conclusion

To the best of our knowledge, this is the first study to examine the potential mediating role of adherence to lifestyle recommendations in the relationship between affective temperaments and infertility treatment success. Our results suggest that certain affective temperaments predict dietary adherence, and greater adherence to the recommended diet may help increase the chances of successful infertility treatment. Since adherence to diet is a modifiable risk factor of infertility treatment success, screening for affective temperaments may help to identify potentially adherent and high-risk non-adherent patient groups before the onset of treatment and offer patient-tailored mental health support or interventions, which may help increase the chances of a successful pregnancy and live birth in women undergoing IVF treatment.

## Data Availability

The data that support the findings of this study are available on request from the corresponding author.
